# Converting *Blastocrithidia Nonstop*, a Trypanosomatid With Non‐Canonical Genetic Code, Into a Genetically‐Tractable Model

**DOI:** 10.1111/mmi.15365

**Published:** 2025-04-09

**Authors:** Arnau Galan, Natalya Kraeva, Kristína Záhonová, Anzhelika Butenko, Alexei Yu Kostygov, Zdeněk Paris, Jiří Pergner, Claretta Bianchi, Fadel Fakih, Andreu Saura, Julius Lukeš, Vyacheslav Yurchenko

**Affiliations:** ^1^ Life Science Research Centre, Faculty of Science University of Ostrava Ostrava Czechia; ^2^ Institute of Parasitology, Biology Centre, Czech Academy of Sciences České Budějovice Czechia; ^3^ Department of Parasitology, Faculty of Science Charles University Praha Czechia; ^4^ Division of Infectious Diseases, Department of Medicine University of Alberta Edmonton Alberta Canada; ^5^ Faculty of Science University of South Bohemia České Budějovice Czechia; ^6^ Zoological Institute, Russian Academy of Sciences St. Petersburg Russia

**Keywords:** codon reassignment, CRISPR‐Cas9, genetic code, model organism, trypanosomatids

## Abstract

*Blastocrithidia nonstop* is a protist with a highly unusual nuclear genetic code, in which all three standard stop codons are reassigned to encode amino acids, with UAA also serving as a sole termination codon. In this study, we demonstrate that this parasitic flagellate is amenable to genetic manipulation, enabling gene ablation and protein tagging. Using preassembled Cas9 ribonucleoprotein complexes, we successfully disrupted and tagged the non‐essential gene encoding catalase. These advances establish this single‐celled eukaryote as a model organism for investigating the malleability and evolution of the genetic code in eukaryotes.

## Graphical abstract

*Blastocrithidia* represents a very small group of species, in which all 3 stop codons are reassigned to encode amino acids with only one of them serving a dual role of a sense and a stop codon simultaneously. Here we demonstrated for the first time that *B. nonstop* can be genetically modified. *Blastocrithidia nonstop* has been turned into a genetically tractable model organism.

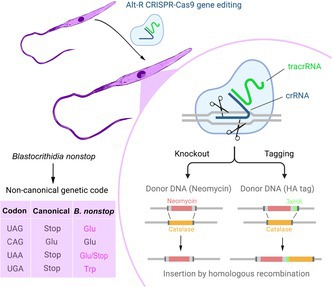

## 1 Introduction

The family Trypanosomatidae (phylum Euglenozoa) is a group of obligatory parasitic protists with a wide host range that includes insects, vertebrates, plants, and even ciliates (Kostygov et al. 2021). These flagellates are non‐taxonomically subdivided into monoxenous and dixenous species: monoxenous trypanosomatids develop in a single host (most commonly, an insect), while their dixenous kin switch between two hosts (Frolov et al. 2021; Lukeš et al. 2018). Because of their medical (*Leishmania* and *Trypanosoma* spp.) and economical (*Phytomonas* spp.) importance, research into this family has been traditionally restricted to the dixenous parasites (Stuart et al. 2008; Bruschi and Gradoni 2018; Jaskowska et al. 2015). Nevertheless, in the past decades, monoxenous trypanosomatids have started to draw increasing attention due to their negative effect on insect populations (Barribeau and Schmid‐Hempel 2013; Hamilton et al. 2015), capacity to opportunistically infect vertebrates, including humans (Maslov et al. 2013; Rogerio et al. 2023), and their pivotal role in inferring and understanding the evolutionary pathways leading to dixeny (Kostygov et al. 2024).

Numerous key molecular mechanisms, such as RNA editing, polycistronic transcription, extensive *trans‐*splicing, GPI anchoring of membrane proteins, and complex mitochondrial DNA (Clayton 2019; Aphasizheva et al. 2020; Michaeli 2011; Lukeš et al. 2002) were initially discovered and characterized in trypanosomatids. They were later found to occur, to varying extents, in a wide range of eukaryotes (Lukeš et al. 2018, 2023). Moreover, due to their cheap axenic cultivation, amenability to forward and reverse genetics, availability of high‐quality genome assemblies and annotations, and the ability of some of these species to infect humans, trypanosomatids now belong to the best studied protists, with outsized importance for our understanding of numerous evolutionary adaptations.

The number of trypanosomatid species with sequenced, assembled, and annotated genomes that are amenable to genetic manipulations and, thus, considered model organisms, remains rather limited (Yurchenko et al. 2021). The bottleneck here is not at the level of genomic sequencing or analysis (Albanaz et al. 2023), but rather at the level of adaptability of different established techniques to a particular species or an isolate. The approach most commonly used within the last couple of years relies on different variations of the CRISPR‐Cas9 technology (Beneke and Gluenz 2019; Yagoubat et al. 2020; Lander and Chiurillo 2019). Nevertheless, classical methods based on homologous recombination or episomal expression are still broadly used (Kim et al. 2013; Feng et al. 2018; Kraeva et al. 2014; Pyrih et al. 2023).

Recently, members of the trypanosomatid genus *Blastocrithidia* were shown to be equipped with arguably one of the most bizarre variants of the nuclear genetic code, in which all three standard stop codons are reassigned to encode amino acids, with one of them (UAA) also serving as a sole stop codon (Záhonová et al. 2016; Kachale et al. 2023). Reassignment of all three stop codons in the nuclear genome appears to be highly restricted in nature. In addition to several *Blastocrithidia* species, it has only been documented in ciliates of the genera *Condylostoma* and *Parduczia*, and marine syndineans of the genus *Amoebophrya* (Swart et al. 2016; Heaphy et al. 2016; Bachvaroff 2019). In *Blastocrithidia nonstop*, the iconic representative of the genus *Blastocrithidia*, the frequency of in‐frame stop codons reversely correlates with gene expression level (Kachale et al. 2023), although this does not seem to affect the general metabolic capacity of this flagellate (Opperdoes et al. 2024). Moreover, its mitochondrial genome and transcriptome appear to be insulated from the nuclear code reassignment (Afonin et al. 2024). It has been widely speculated that the altered genetic code may serve as a barrier protecting its bearers from viral infections and gene transfers (Holmes 2009; Taylor et al. 2013; Lajoie et al. 2013), and this prediction, albeit with low confidence due to the small number of available isolates, has been supported by evidence from *Blastocrithidia* spp. (Grybchuk et al. 2024).

Although departures from the standard (or canonical) genetic code have so far been mostly documented in the mitochondria (Žihala et al. 2020; Macher et al. 2023), more extensive sequencing of understudied eukaryotic lineages revealed a growing number of non‐canonical codes in the nuclear genomes of protists (Pánek et al. 2017; Rotterová et al. 2024), with the dominant position of ciliates (Swart et al. 2016; Heaphy et al. 2016; Gaydukova et al. 2023; McGowan et al. 2024). However, except for a unique and still poorly understood amino acid reassignment described in several yeast lineages (Ó Cinnéide et al. 2024), all known non‐canonical genetic codes evolved in protists that are either unavailable in culture, cultivable only with diverse bacteria, and/or for which methods of genetic manipulation have not yet been established. This is a major limitation for studying alterations of the genetic code in vivo, as almost no information other than of descriptive nature can be obtained from these organisms. However, *B. nonstop* is unique among the eukaryotes with non‐canonical code as it is closely related to the iconic trypanosomatids, for which a wide range of methods of forward and reverse genetics have been established (Matthews 2015). As demonstrated herein, we were able to generate a stable gene ablation and tagging in this flagellate, which, to the best of our knowledge, represents the first genetic modification of a eukaryote with an altered nuclear genetic code. Moreover, it bears a promise to breach the experimental limitations currently imposed by these organisms. Indeed, converting the idiosyncratic *B*. *nonstop* into a model organism will offer an unprecedented opportunity to dissect and manipulate the molecular mechanisms governing departures from the standard genetic code. It may even serve as a platform for exploring what is possible in the evolutionary diversification of the genetic code, following the maxim “normals teach us rules, outliers teach us laws”.

## 2 Materials and Methods

### 2.1 Cell Culture and Species Validation

The culture of *B. nonstop* (isolate P57) (Kachale et al. 2023) was maintained in Schneider's *Drosophila* medium (VWR/Avantor, Radnor, USA) supplemented with 10% heat‐inactivated Fetal Bovine Serum (Biowest, Nuaillé, France), 100 units/mL of penicillin, 100 μg/mL of streptomycin (VWR/Avantor), and 8 μg/mL of hemin (Sigma Aldrich/Merck, Saint Louis, USA) at 25°C, passaging it once every 2 weeks. In our hands, the *B. nonstop* culture reached a cell density of about 1.5 × 10^6^ cells/ml. The species identity was validated by amplifying and sequencing its 18S rRNA gene with primers S762 and S763 (Yurchenko et al. 2016).

### 2.2 Transfection and Selection of *B. nonstop*

#### 2.2.1 Antibiotic Sensitivity

The IC_50_ values for selected antibiotics that are routinely used in trypanosomatid research were determined as described previously (Chmelová et al. 2021). The following antibiotics were tested in a range from 0 to 500 μg/mL: blasticidin (Sigma Aldrich/Merck), geneticin G418 (Thermo Fisher Scientific, Waltham, USA), hygromycin (Carl Roth, Karlsruhe, Germany), and puromycin (Thermo Fisher Scientific).

#### 2.2.2 Design of crRNA Sequences

The sequences of the 20 nucleotide (nt)‐long crRNAs were designed using the Eukaryotic Pathogen CRISPR guide RNA/DNA Design Tool EuPaGDT (Peng and Tarleton 2015) with default parameters (SpCas9: gRNA length 20; PAM: NGG; off‐target PAM: NAG, NGA, where N denotes any nucleotide).

#### 2.2.3 Preparation of a Donor Sequence for Gene Ablation

**FIGURE 1:**
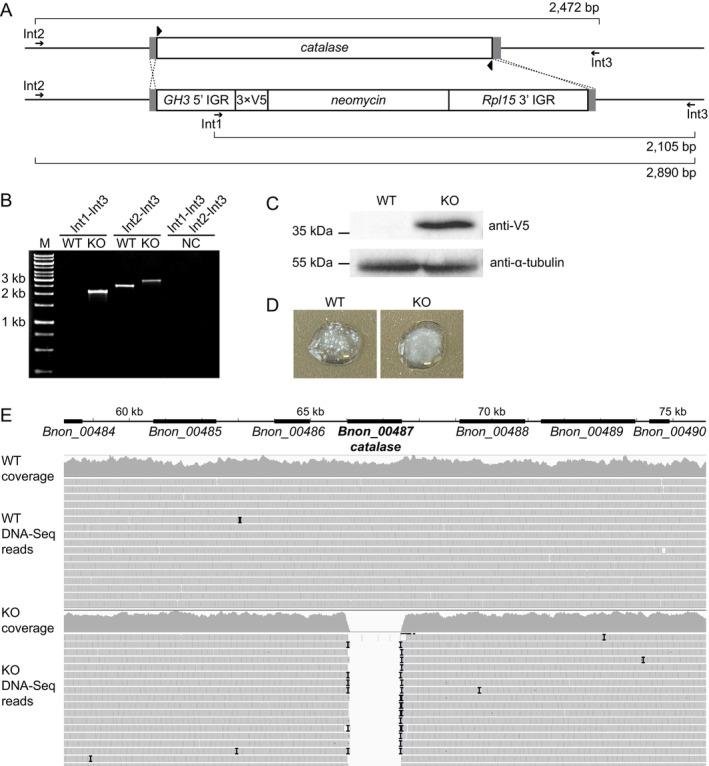
Ablation of *catalase* in *B. nonstop*. (A) Scheme of the knock‐out experiment. Homology arms are shown in grey; crRNA sites are marked by black triangles. Primers used for PCR validation and sizes of the corresponding amplification product are shown. (B) PCR validation of the successful ablation of both *catalase* alleles. M is 1 kb‐ladder molecular weight marker; WT, KO and NC stand for the wild‐type, knock‐out, and negative (no template) control, respectively. (C) Validation of the V5‐neomycin expression by western blotting. Molecular weight sizes in kDa are indicated on the left; probing with anti‐tubulin antibody served to control loading. (D) Activity test validation of the successful ablation of both *catalase* alleles. (E) Genomic confirmation of the successful ablation of both *catalase* alleles. Chromosomal locus of the *Bnon_00487* and surrounding loci is shown on the top. Symbol I indicates indels.

An expression cassette for the V5‐tagged neomycin‐kanamycin phosphotransferase gene (*neomycin*, Figure 1A) providing resistance against G418 and targeting the *B. nonstop* gene encoding *catalase* (*Bnon_00487*, GenBank accession number MK934828) was created by fusion PCR (Merritt and Stuart 2013). Flanking UTRs (5′ UTR of a putative member of the glycoside hydrolase family 3, *Bnon_04072* and 3′ UTR of a putative L15 ribosomal protein, *Bnon_00321*) were chosen based on their expression levels (RPKM [Reads Per Kilobase per Million mapped reads] values 33 and 4066 for *GH3* and *Rpl15*, respectively (Kachale et al. 2023)). In addition, these genes were found in different long contigs, thus excluding the potential appearance of large deletions if intra‐chromosomal recombination events happen. These UTR sequences were amplified using primer pairs F1‐KO/F2‐KO and F5‐KO/F6‐KO (Figure S1, Table S1) for the 5′‐and 3′ UTRs, respectively. The sequence of the 3 × V5‐tagged neomycin‐kanamycin phosphotransferase gene was amplified from the plasmid p57‐V5 + NeoR (GenBank: MN047315) (Faktorová et al. 2020) using primers F3‐KO and F4‐KO and fused with UTRs using primers F7‐KO and F8‐KO that included 30‐nt homology regions with sequences of the catalase locus (Figure S1, Table S1).

#### 2.2.4 Preparation of a Donor Sequence for Gene Tagging

**FIGURE 2:**
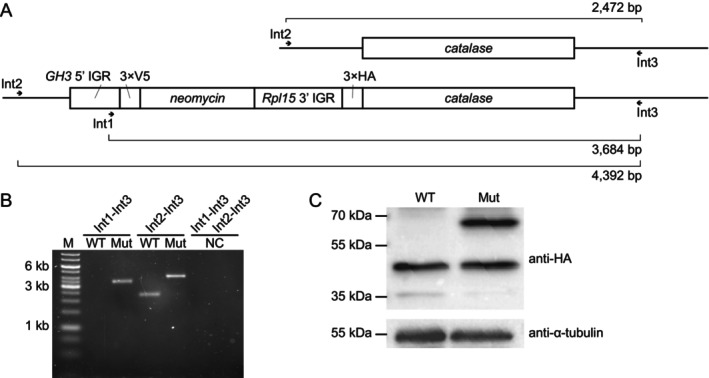
Tagging of catalase in *B. nonstop*. (A) Scheme of the tagging experiment. Primers used for PCR validation and sizes of the corresponding amplification product are shown. (B) PCR validation of the successful tagging of catalase. M is 1 kb‐ladder molecular weight marker; WT and Mut stand for the wild‐type and tagged alleles, respectively, NC stands for the negative (no template) control. (C) Validation of the HA‐tagged catalase expression by western blotting. Molecular weight sizes in kDa are indicated on the left; abbreviations WT and Mut are as above; probing with anti‐tubulin antibody served to control loading. The non‐specific lower band after probing with anti‐HA antibody served as an additional loading control.

An expression cassette for the 3 × V5‐tagged neomycin followed by the intergenic region and 3 × HA‐tagged N‐terminus of the *B. nonstop* catalase was created by fusion PCR (Figure 2A). The 3′ arm is homologous to the first 30 nt following the ATG codon of the *catalase* gene.

#### 2.2.5 Preparation of Cas9 Ribonucleoprotein Complexes

Alt‐R CRISPR‐Cas9 nuclease V3, crRNAs and tracrRNA (Table S1) were synthesized by Integrated DNA Technologies (IDT, Coralville, USA). The Cas9 ribonucleoprotein was prepared as described previously (Nomura et al. 2020). In brief, equal amounts of TracrRNA and crRNA (100 μM each) were mixed and heated at 95°C for 5 min to disrupt potential secondary structures, followed by cooling to 20°C at the rate of −0.1°C/s. Next, the Cas9 protein (62 μM) was added to the mixture and the reaction was incubated at 20°C for 15 min.

#### 2.2.6 Transfection

Forty million cells were electroporated with 5 μg of Cas9 ribonucleoprotein along with 5 μg of donor DNA using one pulse of the X‐033 program in the Amaxa Nucleofector IIb (Lonza, Basel, Switzerland). Cells were recovered in antibiotic‐free cultivation medium for 16 h at 25°C and then selected for 21 days in the presence of 100 μg/mL of G418.

### 2.3 Validation of Gene Ablation and Tagging in *B. nonstop*

*Catalase* gene ablation was validated by: (i) The diagnostic PCR with primers Int1, Int2, and Int3 (Figure 1, Table S1), (ii) western blotting using horseradish peroxidase‐conjugated monoclonal anti‐V5 antibody (catalog number R96125, Thermo Fisher Scientific) and monoclonal anti‐α‐tubulin antibody (catalog number T9026, Sigma Aldrich/Merck) for the loading control diluted 1:1000 and 1:5000, respectively, and (iii) the catalase activity test as described previously (Sádlová et al. 2021). In addition, total genomic DNA of the catalase^−/−^ cells was isolated and sequenced at Macrogen Europe (Amsterdam, Netherlands) as described previously (Albanaz et al. 2021). Raw DNA‐seq reads were adapter and quality trimmed using BBDuk v. 38.98 (Bushnell et al. 2017) with the following parameters: qtrim = *r*l; trimq = 20; ktrim = *r*; *k* = 22; mink = 11; hdist = 2; tpe; tbo. Trimmed reads were mapped onto the reference *B. nonstop* genome (Kachale et al. 2023) using Bowtie2 v. 2.5.2 (Langmead and Salzberg 2012) and SAMtools v. 1.18 (Li et al. 2009), and visualized using IGV v. 2.8.2 (Robinson et al. 2011).

The tagging of catalase was validated by diagnostic PCR with primers Int1, Int2, and Int3 (Figure 2, Table S1) and western blotting using monoclonal anti‐HA antibody (catalog number 26183, Thermo Fisher Scientific) diluted 1:1000 followed by rabbit anti‐mouse IgG antibody (catalog number SAB5600195, Sigma Aldrich/Merck) diluted 1:10,000. Western blotting with anti‐tubulin antibody served as a loading control.

## 3 Results and Discussion

For the “proof of principle” that *B. nonstop* can be genetically modified, we selected a gene encoding catalase. The choice was determined by three factors: (i) the genomes of most trypanosomatids do not encode catalase, so the enzyme appears to be dispensable (Opperdoes et al. 2016; Škodová‐Sveráková et al. 2020); (ii) the ablation of *catalase* in another trypanosomatid (*Leptomonas seymouri*) had a very limited, if any, effect in vitro and in the experimental infection of the insect host of this parasite (Chmelová et al. 2024); (iii) the gene is expressed at the intermediate level in *B. nonstop* as compared to other genes (ranked 4397 out of 7258 transcripts based on the RPKM value (Kachale et al. 2023)).

First, we determined the IC_50_ values for four antibiotics that are routinely used in trypanosomatid research. The values were: 13.5 ± 4.5; 31 ± 6; 13.5 ± 1.5, and 13 ± 1 μg/mL for blasticidin, hygromycin, G418, and puromycin, respectively. These are ranges comparable with other trypanosomatids and, hence, applicable for selection purposes in follow‐up studies.

For knock‐out, we decided to adopt a recently developed highly efficient method of genetic modification that relies on the preassembled Cas9 ribonucleoprotein complexes (Nomura et al. 2020, 2019) to ablate catalase in *B. nonstop*. In the resultant cell line (hereafter named KO) (Figure 1A), a complete deletion of both alleles of *catalase* was documented. It was confirmed on the DNA level by diagnostic PCR (Figure 1B), on the protein level by western blotting (Figure 1C) and the catalase activity test (a production of molecular oxygen that manifests itself by “bubbling”) (Figure 1D). Finally, total genomic DNA obtained from the *catalase* ^−/−^ *B. nonstop* cells was sequenced, and the reads were mapped onto the wild‐type reference genome assembly (Kachale et al. 2023), confirming a successful elimination of the target gene in the KO cell line (Figure 1E). Our expectation that this peculiar gene may be dispensable under the cultivation conditions proved correct. Next, we tagged *B. nonstop* catalase with an HA tag using a similar strategy (Figure 2) and validated it by diagnostic PCR (Figure 2B) and western blotting (Figure 2C). The target protein was successfully tagged, confirming the functionality of yet another type of genetic manipulation.

In our opinion, the most important aspect of the presented study is that we were able to demonstrate that a protist with a non‐canonical nuclear genetic code that departed from the classical molecular biology textbooks in a rather extreme fashion is genetically tractable. The presented results potentially open a whole new research direction. Indeed, major efforts are currently being invested in compressing the standard genetic code, namely, generating an artificial genetic code in living organisms that would allow liberation of certain codons for the incorporation of non‐canonical amino acids (Mukai et al. 2017). In groundbreaking studies performed in *Escherichia coli* , selected codons specifying the insertion of a given amino acid, for which other codons remained available, were systematically replaced across the genome (Fredens et al. 2019), followed by an indispensable elimination of their cognate tRNAs (Robertson et al. 2021; Zürcher et al. 2022). The very first genetic code contraction has been achieved by replacing all the UAG stop codons with UAA, thus allowing potential repurposing of the former codon (Lajoie et al. 2013). As a matter of fact, a very similar reassignment occurred in *B. nonstop*, as this parasitic protist uses UAA as its sole termination codon (Kachale et al. 2023). Converting this species into a genetically tractable model will allow exciting further in vivo experiments, which might include manipulations with stop codons, amino acyl‐tRNA synthetases, and release factors in a eukaryote in which genetic code contractions have been achieved purely by evolutionary processes that likely occurred millions of years ago.

## Author Contributions

**Arnau Galan:** investigation, writing – review and editing. **Natalya Kraeva:** investigation, validation, methodology, writing – review and editing, data curation. **Kristína Záhonová:** investigation, validation, formal analysis, writing – review and editing. **Anzhelika Butenko:** investigation, validation, formal analysis, writing – review and editing. **Alexei Yu Kostygov:** investigation, writing – review and editing, validation, formal analysis. **Zdeněk Paris:** writing – review and editing, data curation. **Jiří Pergner:** data curation, writing – review and editing. **Claretta Bianchi:** investigation, data curation, writing – review and editing. **Fadel Fakih:** validation, data curation, writing – review and editing. **Andreu Saura:** writing – review and editing, data curation, formal analysis, investigation. **Julius Lukeš:** funding acquisition, writing – review and editing, data curation, supervision, validation, resources. **Vyacheslav Yurchenko:** conceptualization, funding acquisition, writing – original draft, writing – review and editing, visualization, validation, methodology, formal analysis, project administration, supervision.

## Conflicts of Interest

The authors declare no conflicts of interest.

## Supporting information


**Data S1.** Supporting Information.

**Table S1.** Supporting Information.

**Figure S1.** Amplification of the DNA fragments used for the catalase ablation and tagging in *B. nonstop*. Annealing positions of the used primers and expected fragment sized are indicated. All other abbreviations are as in Figure 1.

## Data Availability

The data that support the findings of this study are available in NCBI at https://www.ncbi.nlm.nih.gov/datasets/genome/, reference number ASM2855474. These data were derived from the following resources available in the public domain: ‐ NCBI Genome assembly, https://www.ncbi.nlm.nih.gov/datasets/genome/GCA_028554745.1/.
