# Supplementary material for: Converting Blastocrithidia Nonstop, a Trypanosomatid With Non‐Canonical Genetic Code, Into a Genetically‐Tractable Model
Source: Mol Microbiol. 2025 Apr 9;123(6):586–92. doi: 10.1111/mmi.15365 (PMC12152300; doi:10.1111/mmi.15365)
Supplement: Supplementary file 1 — Data S1. Supporting Information. [file MMI-123-586-s002.docx]

A scanning electron micrograph of Blastocrithidia nonstop rendered using diffferent lookup tables. This kinetoplastid parasite reads through stop codons and can now be genetically modified."
