# Supplementary figures and images for: Converting Blastocrithidia Nonstop, a Trypanosomatid With Non‐Canonical Genetic Code, Into a Genetically‐Tractable Model
Source: Mol Microbiol. 2025 Apr 9;123(6):586–92. doi: 10.1111/mmi.15365 (PMC12152300; doi:10.1111/mmi.15365)

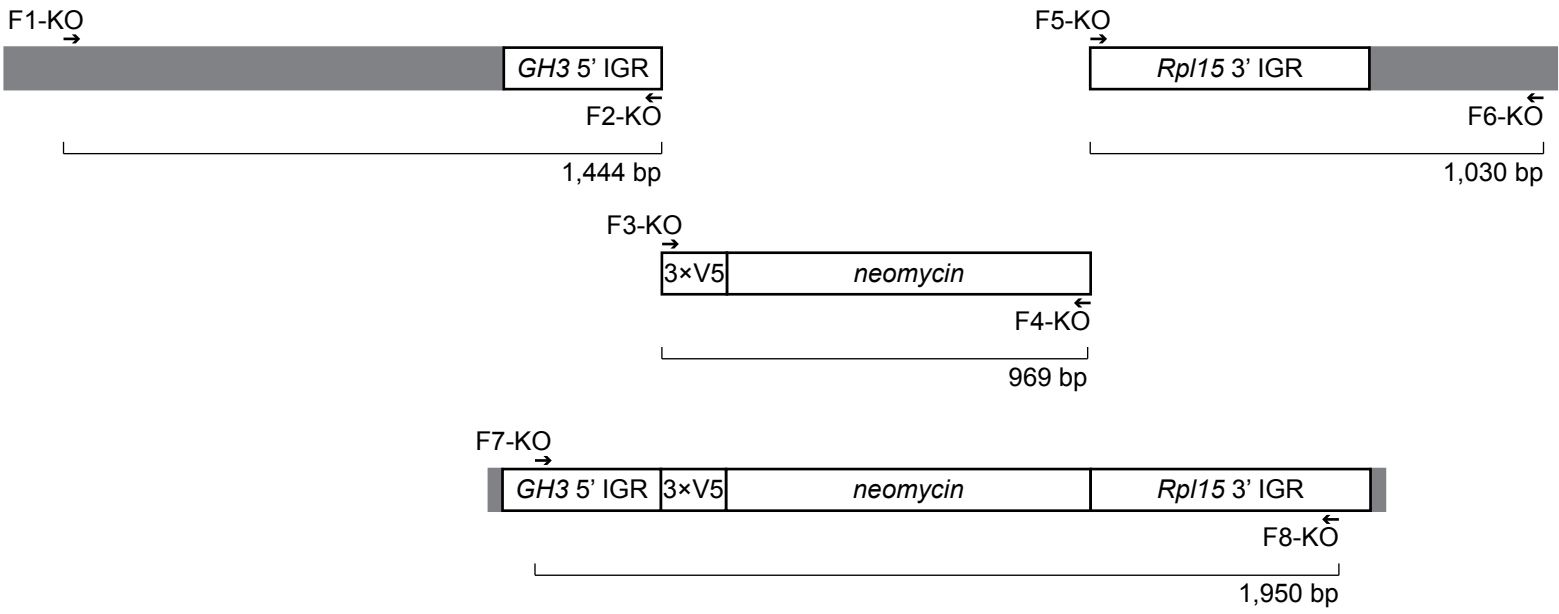

Supplement: Supplementary file 3 — Figure S1. Amplification of the DNA fragments used for the catalase ablation and tagging in B. nonstop. Annealing positions of the used primers and expected fragment sized are indicated. All other abbreviations are as in Figure 1. [file MMI-123-586-s003.pdf]
